# Estrogen Attenuates Hypoxia-Induced TRPV1 Activation and Calcium Overload via HIF-1α Suppression in MCF-7 and CHO Cells

**DOI:** 10.3390/ijms262211110

**Published:** 2025-11-17

**Authors:** Bilal Çiğ

**Affiliations:** 1 Department of Physiology, Medical Faculty, Kirsehir Ahi Evran University, 40100 Kirsehir, Türkiye; bilal.cig@ahievran.edu.tr; 2 Department of Neuroscience, Institute of Health Sciences, Kirsehir Ahi Evran University, 40200 Kirsehir, Türkiye

**Keywords:** hypoxia, HIF-1α, 17β-estradiol, TRPV1, calcium signaling, MCF-7, CHO

## Abstract

Hypoxia is a major global health concern, particularly in premature infants and cancer, where it promotes intracellular calcium accumulation and cell death. The transient receptor potential vanilloid 1 (TRPV1) channel has been implicated in calcium dysregulation and oxidative stress under hypoxic conditions, while estrogen (17β-estradiol, E_2_) is known to modulate TRPV1 activity and redox balance. This study aimed to investigate the impact of E_2_ on TRPV1 expression, hypoxia-inducible factor-1α (HIF-1α), and calcium signaling in MCF-7 breast cancer cells (ERα-positive) and TRPV1-transfected CHO cells (ERα-negative). Four experimental groups were established: normoxia, E_2_, hypoxia, and hypoxia + E_2_. Hypoxia was induced by CoCl_2_ (200 µM, 24 h), while E_2_ treatment was applied at 10 nM for 24 h. Western blot analysis revealed that both TRPV1 and HIF-1α expression were upregulated under hypoxia but significantly reduced by E_2_. Fura-2 fluorescence assays revealed that hypoxia increased cytosolic Ca^2+^ levels, whereas E_2_ reversed this elevation. Moreover, TRPV1 activation by capsaicin induced marked Ca^2+^ influx under hypoxia, which was attenuated by E_2_ treatment. These findings demonstrate that E_2_ mitigates hypoxia-induced toxicity by modulating TRPV1-mediated Ca^2+^ signaling and HIF-1α expression, underscoring the protective role of E_2_ and identifying TRPV1 as a potential therapeutic target in estrogen-responsive tumors.

## 1. Introduction

Oxygen serves as the terminal electron acceptor in aerobic cells, and its reduction (hypoxia) profoundly reprograms cellular metabolism, transcriptional networks, and ion channel functions [1]. At the core of the hypoxic response is hypoxia-inducible factor-1α (HIF-1α), which drives a metabolic shift from oxidative phosphorylation toward glycolysis by upregulating glycolytic enzymes and glucose transporters, while simultaneously activating a broad array of genes involved in angiogenesis and tissue remodeling [2]. Yet, this adaptation extends beyond the HIF family: nuclear receptors such as estrogen-related receptor-α (ERRα) can cooperate with HIF to intensify metabolic reprogramming or regulate HIF-independent targets [1]. Energy deprivation and oxygen stress also disrupt membrane potential, endoplasmic reticulum–mitochondrial calcium coupling, and cytoskeletal integrity, adding further complexity to the hypoxic phenotype [3,4,5].

Such hypoxia-driven remodeling of energy metabolism and intracellular signaling provides a foundation for altered ion homeostasis and stress adaptation in many cell types. In particular, the intersection between transcriptional control (HIF/ERRα) and calcium regulation defines whether cells survive or succumb to hypoxic stress. Estrogen receptor-α (ERα) is a key determinant of proliferation and survival, particularly in luminal ER-positive breast cancer (e.g., MCF-7). Increasing evidence indicates that hypoxia engages in multifaceted crosstalk with ERα, reshaping its cistrome, sustaining certain targets in a hormone-independent manner, and thereby altering sensitivity to endocrine therapy, while also inducing context-specific and cell type–dependent changes in ERα expression and activity [6,7].

Post-translational regulators, including members of the PARP family, further fine-tune this signaling by suppressing ERα activity, suggesting multiple layers of control under stress conditions [8,9]. Ion channels represent an additional functional output of this network. TRPV1, a non-selective cation channel gated by protons, heat, and capsaicin, is highly permeable to calcium and shapes intracellular signaling at both the plasma membrane and organelle interfaces [3,10]. In hypoxia/reoxygenation models, excessive TRPV1 activation induces Ca^2+^ overload and stress phenotypes, whereas inhibition of the channel provides protection [5,11]. Estradiol has been shown to enhance TRPV1 expression and responsiveness in ERα-positive cells, suggesting that the estrogenic milieu may lower the threshold for TRPV1 activation, thereby eliciting rapid, non-genomic effects on calcium signaling [12,13].

These observations suggest that the Ca^2+^ signaling machinery is a major downstream effector of hypoxia and estrogen interaction. The stabilization of HIF-1α under hypoxia may transcriptionally or post-transcriptionally sensitize TRPV1 channels, while estrogenic signaling can provide a compensatory brake on this calcium influx. Hypoxia-driven stabilization of HIF-1α may thus prime TRPV1 into a “ready-to-activate” state, while estrogenic signaling modulates this sensitivity, establishing a convergent Ca^2+^ axis [3,14,15]. Importantly, the interplay between hypoxia and estrogen signaling is not restricted to tumor cells. In microglial models, ERRα confers homeostatic protection during hypoxic injury, while E_2_ reprograms iron metabolism via HIF-1α upregulation independently of the IRP/hepcidin pathway, highlighting the tissue-specific breadth of nuclear receptor–HIF interactions [16,17]. Conversely, in ER(+) breast cancer, hypoxia initiates a HIF-driven transcriptional program that strengthens the foundation for endocrine resistance [7,18,19,20].

Collectively, these studies outline a mechanistic framework in which HIF-1α stabilization, ERα/ERRα signaling, and TRPV1-dependent calcium flux are functionally integrated. This triad governs redox balance, mitochondrial activity, and ultimately cell fate under oxygen deprivation. Clarifying how estrogen modulates this axis may reveal novel targets to counteract hypoxia-induced calcium overload and therapy resistance in ERα-positive cancers. The present study investigates the effects of CoCl_2_-induced hypoxia on HIF-1α and TRPV1 expression, as well as associated calcium dynamics, in MCF-7 cells and TRPV1 transfected CHO models. By integrating these experimental platforms, we aim to delineate the functional continuity of this axis and clarify its role in hypoxia-driven cellular adaptation.

## 2. Results

### 2.1. Effects of Hypoxia and Estrogen on Intracellular Calcium Dynamics in MCF-7 and CHO Cells

**Figure 1:**
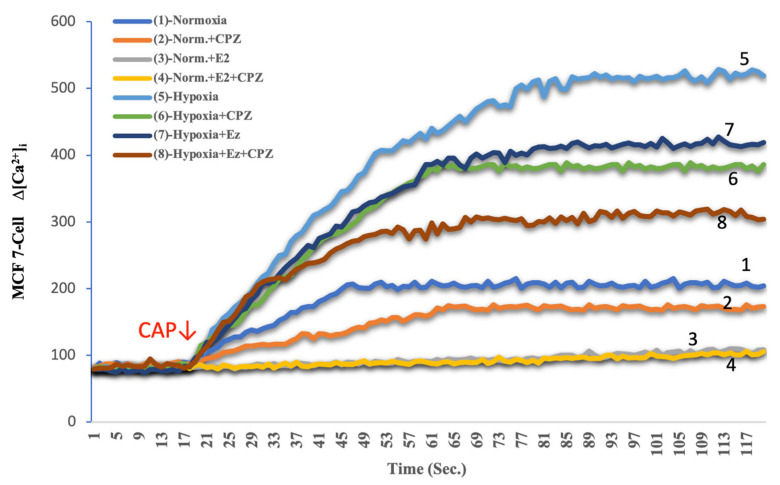
Effects of E_2_ on intracellular Ca^2+^ dynamics in MCF-7 cells under hypoxic and normoxic conditions (mean ± SEM, *n = 3*). Fluorescence-based Ca^2+^ imaging was performed using Fura-2 AM. The arrow indicates the time point of capsaicin (CAP, 1 μM) application to activate TRPV1 channels. Hypoxia markedly elevated intracellular Ca^2+^ levels compared with normoxia (trace 5 vs. 1), confirming enhanced TRPV1 activity under low-oxygen conditions. E_2_ treatment significantly attenuated this Ca^2+^ rise (trace 7 vs. 5), and the TRPV1 antagonist capsazepine (CPZ, 10 μM) abolished the CAP-induced signal in both normoxic and hypoxic states (traces 2, 4, 6, and 8). These data indicate that hypoxia sensitizes TRPV1-mediated calcium entry, whereas estradiol counteracts this effect, supporting its protective role against hypoxia-induced calcium overload in MCF-7 cells.

**Figure 2:**
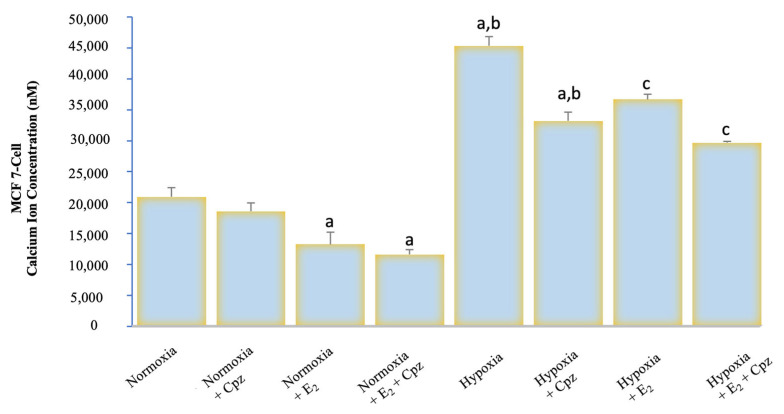
Quantitative analysis of intracellular Ca^2+^ concentration in MCF-7 cells exposed to hypoxia and treated with E_2_ and/or the TRPV1 antagonist capsazepine (CPZ) (mean ± SEM, *n = 3*). Hypoxia markedly increased intracellular Ca^2+^ concentration compared with normoxia (*p* < 0.001), indicating enhanced TRPV1 activity under low-oxygen conditions. E_2_ treatment significantly reduced Ca^2+^ accumulation (*p* < 0.001 vs. hypoxia), demonstrating its suppressive effect on TRPV1-mediated calcium entry. Co-application of CPZ further diminished this response, confirming channel specificity. Statistical significance: ^a^ *p* < 0.001 vs. Normoxia; ^b^ *p* < 0.001 vs. E_2_; ^c^ *p* < 0.001 vs. Hypoxia.

**Figure 3:**
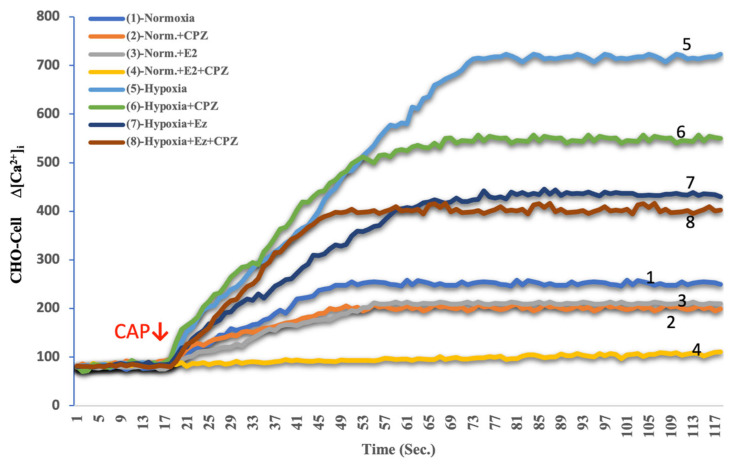
Effects of E_2_ on intracellular Ca^2+^ dynamics in TRPV1-transfected CHO cells under hypoxic and normoxic conditions (mean ± SEM, *n = 3*). These CHO cells were transiently transfected with a full-length human TRPV1 construct to enable functional Ca^2+^ imaging of TRPV1 activity. Fluorescence recordings were obtained using Fura-2 AM, and the red arrow indicates the time of capsaicin (CAP, 1 μM) application to activate TRPV1 channels. Hypoxia markedly enhanced Ca^2+^ influx compared with normoxia (trace 5 vs. 1), consistent with increased TRPV1 responsiveness under low-oxygen stress. Unlike ERα-positive MCF-7 cells, E_2_ treatment did not significantly alter the Ca^2+^ response (trace 7 vs. 5), confirming the absence of estrogen-dependent modulation in ERα-negative CHO cells. Capsazepine (CPZ, 10 μM) suppressed the CAP-evoked signal in all conditions (traces 2, 4, 6, 8), validating TRPV1 specificity. Together, these data demonstrate that hypoxia enhances TRPV1-mediated calcium entry independently of estrogen signaling in CHO-TRPV1 cells.

**Figure 4:**
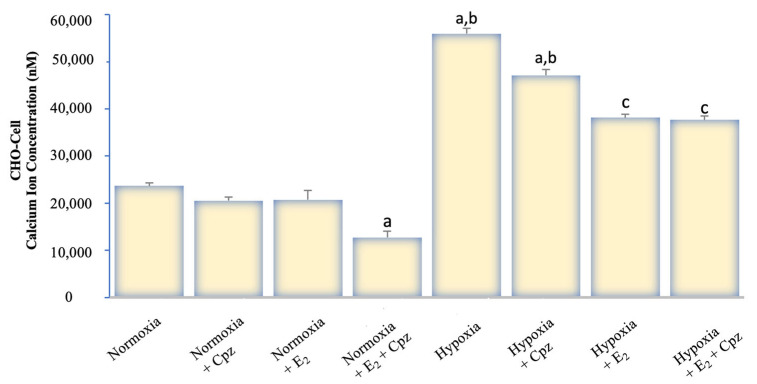
Quantitative analysis of intracellular Ca^2+^ concentration in TRPV1-transfected CHO cells under normoxic and hypoxic conditions following treatment with E_2_ and/or capsazepine (CPZ) (mean ± SEM, *n = 3*). Hypoxia caused a pronounced elevation in intracellular Ca^2+^ levels compared with normoxia (*p* < 0.001), confirming strong TRPV1 activation under low-oxygen stress. In contrast to ERα-positive MCF-7 cells, E_2_ did not significantly modify Ca^2+^ accumulation in CHO-TRPV1 cells, indicating the absence of estrogen-dependent modulation. CPZ treatment markedly reduced Ca^2+^ influx in both normoxic and hypoxic conditions, verifying TRPV1 channel specificity. Statistical significance: ^a^ *p* < 0.001 vs. Normoxia; ^b^ *p* < 0.001 vs. E_2_; ^c^ *p* < 0.001 vs. Hypoxia.

Calcium analysis with Fura-2 AM demonstrated marked differences between experimental groups. Under normoxic conditions, baseline cytosolic Ca^2+^ levels in both MCF-7 and CHO cells remained relatively stable, with modest responses to capsaicin (CAP) stimulation (Figure 1, Figure 2, Figure 3 and Figure 4). Addition of capsazepine (CPZ), a TRPV1 antagonist, further reduced Ca^2+^ influx, confirming TRPV1 dependence of the observed response. E_2_ treatment under normoxia resulted in a significant suppression of Ca^2+^ entry, and the combined E_2_ + CPZ condition produced the lowest Ca^2+^ signals among all normoxic groups (*p* < 0.001 vs. normoxia). Exposure to chemical hypoxia (CoCl_2_, 200 µM, 24 h) induced a robust elevation in cytosolic Ca^2+^ concentration in both MCF-7 and CHO cells. CAP stimulation further potentiated this effect, leading to the highest Ca^2+^ peaks observed across all conditions. Notably, co-treatment with E_2_ significantly attenuated hypoxia-induced Ca^2+^ influx (*p* < 0.001 vs. hypoxia), and this effect was further diminished in the presence of Cpz (Figure 2 and Figure 4). These results indicate that hypoxia primes TRPV1-mediated calcium entry, while estradiol exerts a protective effect by reducing Ca^2+^ overload.

### 2.2. TRPV1 Expression in Response to Hypoxia and Estradiol

**Figure 5:**
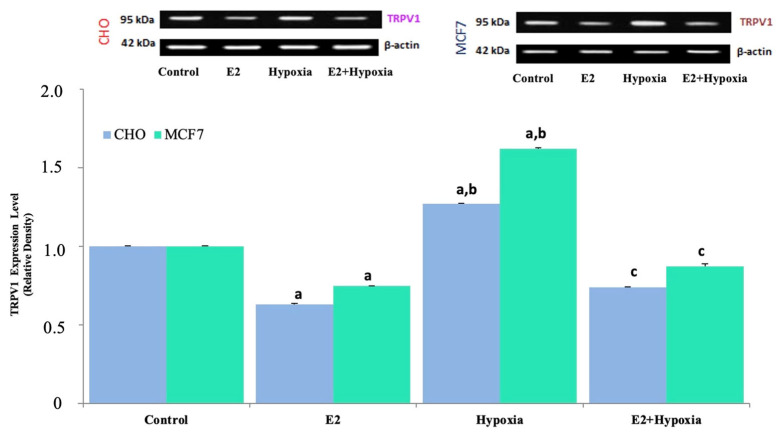
Representative immunoblots and quantitative analysis of TRPV1 protein expression in MCF-7 and TRPV1-transfected CHO cells under normoxic and hypoxic conditions following treatment with E_2_ (mean ± SEM, *n = 3*). β-Actin was used as a loading control for normalization. Hypoxia strongly upregulated TRPV1 expression in both cell lines compared with control (*p* < 0.001), whereas E_2_ alone reduced basal TRPV1 levels relative to normoxia (*p* < 0.001). Co-treatment with E_2_ during hypoxia (E_2_ + Hypoxia) markedly attenuated the hypoxia-induced increase in TRPV1 expression (*p* < 0.001 vs. hypoxia), indicating that E_2_ counteracts hypoxia-driven TRPV1 induction in ERα-positive MCF-7 cells but not in CHO-TRPV1 cells. Statistical significance: ^a^ *p* < 0.001 vs. Control; ^b^ *p* < 0.001 vs. E_2_; ^c^ *p* < 0.001 vs. Hypoxia.

Western blot analyses revealed differential regulation of TRPV1 protein levels across experimental groups (Figure 5). In both MCF-7 and CHO cells, hypoxia significantly upregulated TRPV1 expression compared to normoxic controls (*p* < 0.001). Estradiol treatment alone reduced TRPV1 protein levels (*p* < 0.001 vs. control), and the combination of hypoxia + E_2_ resulted in a partial but significant suppression of TRPV1 expression relative to hypoxia alone (*p* < 0.001). These findings confirm that estradiol downregulates TRPV1 expression even under hypoxic stress conditions, suggesting a modulatory role of estrogenic signaling on TRPV1 activity.

### 2.3. HIF-1α Expression Under Normoxia, Hypoxia, and Estradiol Treatment

**Figure 6:**
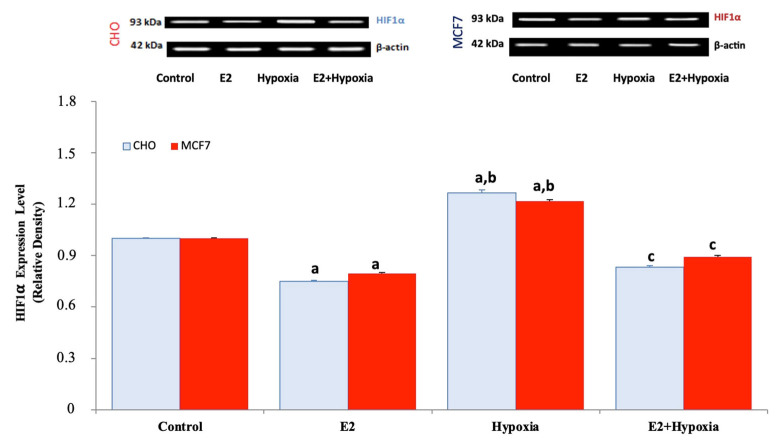
Representative immunoblots and quantitative analysis of hypoxia-inducible factor 1-alpha (HIF-1α) protein expression in MCF-7 and TRPV1-transfected CHO cells under normoxic and hypoxic conditions following E_2_ treatment (mean ± SEM, *n = 3*). β-Actin served as a loading control for normalization. Hypoxia markedly increased HIF-1α expression in both cell lines compared with control (*p* < 0.05), consistent with activation of the canonical hypoxic response. E_2_ alone slightly decreased basal HIF-1α levels relative to control (*p* < 0.05), whereas co-treatment with E_2_ during hypoxia significantly attenuated the hypoxia-induced up-regulation of HIF-1α (*p* < 0.01 vs. hypoxia). These results demonstrate that E_2_ suppresses hypoxia-driven HIF-1α stabilization in ERα-positive MCF-7 cells, whereas this regulatory effect is less pronounced in ERα-negative CHO-TRPV1 cells. Statistical significance: ^a^ *p* < 0.05 vs. Control; ^b^ *p* < 0.01 vs. E_2_; ^c^ *p* < 0.01 vs. Hypoxia. Note: The increase in HIF-1α band intensity in MCF-7 cells is modest but statistically significant when quantified across replicates (*p* < 0.01).

Assessment of HIF-1α expression demonstrated the expected stabilization of the protein in hypoxia-exposed groups (Figure 6). Both MCF-7 and CHO cells displayed significantly elevated HIF-1α levels following CoCl_2_ treatment compared to normoxia (*p* < 0.01). Estradiol treatment alone slightly decreased HIF-1α expression relative to control, whereas the combination of hypoxia + E_2_ significantly attenuated HIF-1α induction compared to hypoxia alone (*p* < 0.01). These results indicate that estradiol not only regulates TRPV1 but also mitigates hypoxia-induced HIF-1α accumulation.

## 3. Discussion

Under chemically induced hypoxia (CoCl_2_), we quantified changes in HIF-1α and TRPV1 protein levels by Western blotting and assessed accompanying intracellular Ca^2+^ dynamics functionally in ERα-positive MCF-7 cells and ERα-negative, TRPV1-transfected CHO cells. Our data show that hypoxia triggered HIF-1α accumulation in both cell types; in parallel, TRPV1 expression increased and capsaicin (CAP)–evoked Ca^2+^ influx was markedly potentiated. In MCF-7 cells, CAP responses were attenuated by E_2_, and in both models the Ca^2+^ rise was abolished by capsazepine, supporting a TRPV1-dependent mechanism. Together, these findings delineate a functional cascade in which hypoxia induces HIF-1α stabilization, leading to increased TRPV1 expression and enhanced calcium influx. This pathway operates with distinct gain settings depending on the presence or absence of ERα and is specifically modulated by an estrogen-mediated brake in MCF-7 cells [7,14,15,16,17,18,19,20,21,22]. HIF-1α induction is the canonical core of hypoxic adaptation and is robustly observed in both true and chemical hypoxia models. Consistent with our blots, hypoxia-mimetics (CoCl_2_) stabilize HIF-1α and elicit broad transcriptional programs across cellular contexts [20,23,24]. In breast cancer cells, CoCl_2_-driven HIF-1α accumulation has been linked to stress signaling (p53, BAX) and phenotypic shifts; depending on dose and exposure, hypoxia can produce bidirectional oscillations between proliferation and apoptosis [25]. Even in pluripotent stem cells, chemical hypoxia can trigger apoptosis through HIF-independent pathways, underscoring that hypoxic outputs are not exclusively HIF-mediated but can engage calcium–redox–mitochondrial axes as well [26]. The hypoxia-associated Ca^2+^ elevation and TRPV1 upregulation we observe directly intersect with this ionic/mitochondrial dimension.

TRPV1 expression and function rose significantly under hypoxia. This aligns with reports that TRPV1 contributes to Ca^2+^ overload, oxidative stress, and cell-fate decisions in hypoxia/reoxygenation paradigms [26,27]. In cardiomyocytes, excessive TRPV1 activation during hypoxia–reoxygenation augments Ca^2+^ entry and compromises viability, whereas antagonism or downregulation is protective [11]. In neural/glial systems, hypoxia-evoked TRPV1 activation feeds into transcriptional modules (e.g., JAK2/STAT3), inflammatory readouts, and Ca^2+^-dependent processes [3,15,27]. Functionally, our observation that CAP-evoked Ca^2+^ signals are amplified by hypoxia and extinguished by capsazepine is fully concordant with this literature [14,27]. ERα status shaped the signaling landscape. Because MCF-7 is ERα(+) and CHO-TRPV1 is ERα(–), the same hypoxic input was interpreted through two distinct signaling architectures. In MCF-7, E_2_ significantly dampened TRPV1-mediated Ca^2+^ spikes, indicating that estrogen can tune TRPV1 sensitivity and counter hypoxic priming [9,15,28,29,30,31]. Prior studies report that estrogen modulates TRPV1 abundance/trafficking in MCF-7, reprograms the channel in an ERα-dependent manner, and that subcellular distribution (plasma membrane vs. ER/Golgi-centered aggregates) correlates with prognosis [12]. Hypoxia, conversely, rewires the ERα cistrome, sustains a subset of targets hormone-independently, and establishes a substrate for endocrine resistance [7]. The observation that E_2_ suppresses CAP responses in MCF-7 cells even under hypoxic conditions suggests that ERα activity is not entirely abolished but instead modulated through ERα–HIF crosstalk in a target-specific manner [13]. In CHO-TRPV1, lacking the ERα layer, the Ca^2+^ phenotype is parsimoniously explained by TRPV1 function and hypoxic priming. Thus, we experimentally separate ER-dependent (MCF-7) and ER-independent (CHO) regimes of the same TRPV1–Ca^2+^ flow.

Emerging evidence further indicates that this hypoxia–estrogen interplay extends to aromatase inhibitor resistance. Letrozole, by blocking estrogen synthesis, can indirectly modify HIF-1α–regulated survival mechanisms even in normoxic environments. In letrozole-resistant breast cancer cells, HIF-1α expression remains constitutively elevated through the HER2–PI3K/Akt/mTOR pathway, driving downstream targets such as BCRP that sustain resistance phenotypes [32]. This “nonhypoxic” activation of HIF-1α reveals that estrogen deprivation itself can reshape hypoxia-related signaling and uncouple it from oxygen dependence. Accordingly, our present finding that estradiol suppresses HIF-1α stabilization and TRPV1 mediated Ca^2+^ influx under hypoxic stress may reflect a physiological counterpart of the same mechanism dysregulated in aromatase inhibitor resistance where loss of estrogenic tone permits persistent HIF-1α signaling and downstream channel activation [32].

Mitochondrial and redox context are integral to these outcomes: Ca^2+^ overload, loss of ΔΨm, ROS escalation, and death programs are tightly coupled [5,13,18]. Work in neuron–astrocyte systems (eOGD-R ± hypothermia) show striking cell-type-specific bioenergetic responses; neurons are more susceptible to energy deprivation and benefit more from hypothermia [33]. Post-hypoxic ROS, lipid peroxidation, and GSH kinetics also diverge by cell type; hypothermia can strongly suppress neuronal ROS while restoring astrocytic GSH [33]. Although our cancer models are non-neural, they reiterate that the hypoxia–Ca^2+^–mitochondria axis is context-sensitive and that TRPV1-driven Ca^2+^ influx can amplify this variability [33,34]. In certain contexts (e.g., osteosarcoma PDT), TRPV1 activation reduces oxygen consumption and HIF-1α accumulation, biasing toward ferroptosis—emphasizing that TRPV1 outputs depend on dose, duration, and metabolic state [35,36]. The mechanistic basis of our chemical hypoxia is well established: Co^2+^ occupies the Fe-binding pocket of PHD enzymes, preventing pVHL recognition and proteasomal degradation of HIF-1α, thereby stabilizing and nuclear-routing HIF-1α [1,17,37]. That said, chemical hypoxia can also activate HIF-independent apoptosis in certain cells [26]. Our prominent TRPV1 Ca^2+^ component supports the notion that CoCl_2_ not only stabilizes HIF-1α but also primes ion-channel repertoires (e.g., TRPV1) to magnify functional outputs [17,26,37]. Notably, CoCl_2_ dose/time produces heterogeneous angiogenic profiles across systems: low doses can transiently elevate VEGF/angiogenic chemokines in some stem-cell models, whereas higher doses favor cytotoxicity [37]. In breast cancer, the HIF–VEGF axis intersects vascular permeability and edema; classic studies demonstrate that hypoxia increases brain vascular leak via VEGF [38]. While we did not assay vascular endpoints, our hypoxic HIF-1α rise and TRPV1 Ca^2+^ axis plausibly interface with microenvironmental angiogenic and trophic interactions [38].

ER-related mechanisms likely operate at two levels in MCF-7: (i) transcriptional crosstalk with HIF shaping endocrine-resistant states [16]; and (ii) rapid, non-genomic tuning of TRPV1 sensitivity via Ca^2+^/kinase cascades [31]. Within the nuclear-receptor layer, ERRα can regulate energy and repair programs under hypoxia; its microglial actions supporting HIF-1α and limiting inflammation illustrate a broader balancing role [1,16]. This raises the testable hypothesis that a portion of the HIF-1α/TRPV1 elevation we observe could be modulated by ERRα; pharmacologic ERRα perturbation and readout of TRPV1 Ca^2+^ would be a logical next step [1,16]. Insights from HT-22 neuronal literature further frame our findings: hypoxia-linked Ca^2+^ dysregulation extends to SOCE components (STIM1/Orai1) and kinases such as CDK5; the Orai1/CDK5 axis drives Tau hyperphosphorylation and neuronal death [28]. While we did not measure Tau in cancer cells, Ca^2+^ loading can rewire kinase networks and participate in HIF–kinase dialogues. In chronic hypoperfusion/ischemia, VEGF-A activates Akt/CREB and modulates autophagy–apoptosis balance [39]. Although VEGF-A in breast cancer is chiefly angiogenic/permeability-related, such growth factors may indirectly influence hypoxic Ca^2+^ kinase tuning [38]. Finally, edaravone and related ROS scavengers mitigate hypoxia–ischemia injury, suggesting that lowering oxidative burden in the Ca^2+^ ROS mitochondria triad could constrain pathological TRPV1-mediated Ca^2+^ influx [34]. Antioxidants combined with Ca^2+^ modulators are therefore rational for rescue experiments in our system.

**Figure 7:**
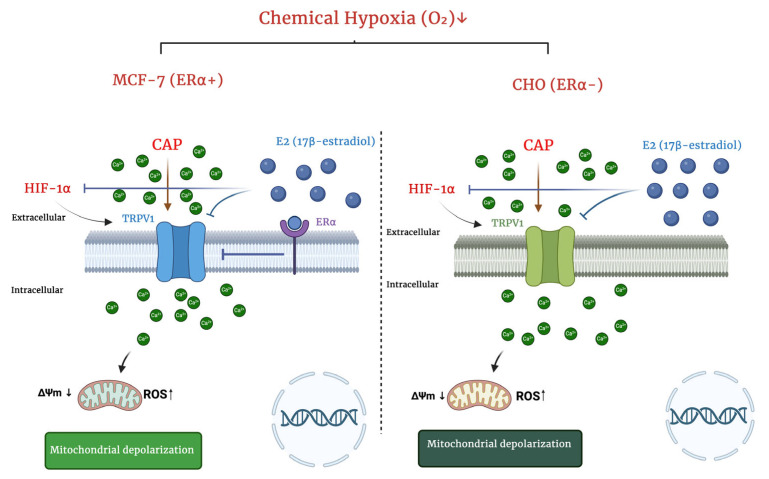
Schematic representation summarizing the ERα-dependent and ERα-independent pathways of hypoxia-induced TRPV1 activation and calcium influx. Under chemical hypoxia (CoCl_2_), HIF-1α stabilization enhances TRPV1 expression and promotes Ca^2+^ entry, leading to mitochondrial depolarization and increased ROS generation. In MCF-7 cells (ERα^+^), E_2_ acts through ERα to attenuate HIF-1α accumulation and suppress TRPV1-mediated Ca^2+^ influx, thereby limiting mitochondrial stress. In contrast, in CHO-TRPV1 cells (ERα^−^), this regulatory brake is absent, resulting in an unrestrained TRPV1 Ca^2+^ HIF-1α feed-forward loop under hypoxia. The figure illustrates how estrogen receptor signaling modulates calcium dynamics and oxidative stress in a cell-type-specific manner. Arrows indicate activation or directional signaling, while flat-ended lines represent inhibition: → Activation/stimulation; ⊣ Inhibition; ↓ or ↑ Increase or decrease; → Ca^2^⁺ influx through TRPV1 channel (BioRender).

Subcellular localization of TRPV1 carries potential clinical meaning: “classical” (plasma membrane/cytoplasmic) distribution correlates with better prognosis, whereas “non-classical” ER/Golgi-centric aggregates associate with poorer survival in breast cancer specimens [12]. Hypoxia perturbs protein folding and membrane trafficking; both TRPV1 quantity and topology may shift. Our functional data (enhanced CAP-evoked currents) imply an increase in conductive, surface-accessible TRPV1 a phenotype distinct from non-classical aggregates. Systematic immunofluorescence co-localization (plasma membrane vs. ER/Golgi markers) under hypoxia would therefore be informative for prognosis and targeting [12]. Methodologically, our dual-model design (ERα(+) MCF-7 and ERα(–) CHO-TRPV1) let us interrogate the same hypothesis across complementary receptor backgrounds, linking Ca^2+^/TRPV1/HIF-1α readouts with E_2_/capsazepine modulation. This multi-pronged approach demonstrates that the hypoxia–HIF → TRPV1 → Ca^2+^ axis is operative and that ERα adjusts its gain [1,2,28] (Figure 7). Limitations include: (i) lack of genetic TRPV1 validation (siRNA/CRISPR), (ii) absence of quantitative imaging of TRPV1 subcellular distribution under hypoxia, and (iii) incomplete dissection of ERRα contributions [1]. Future work should deploy ERRα modulators (e.g., XCT790), ER subtype selective ligands, HIF-1/2α silencing, antioxidants (e.g., edaravone), and TRPV1 antagonism/desensitization to map epistasis and rescue hierarchies [34].

## 4. Materials and Methods

### 4.1. Cell Lines and Culture

The MCF-7 cell line was obtained from PhD Orhan Koçak (Akdeniz University, Antalya, Türkiye) and the CHO cell line was obtained from the Şap Institute (Ankara, Türkiye). ERα-positive human breast adenocarcinoma cells (MCF-7) and Chinese hamster ovary cells transiently transfected to express human TRPV1 (CHO-TRPV1; CHO-K1 background ) were used. MCF-7 cells were maintained in Dulbecco’s Modified Eagle Medium (DMEM, high glucose; Gibco, New York, NY, USA) supplemented with 10% heat-inactivated fetal bovine serum (FBS; Gibco, New York, NY, USA), 1% penicillin/streptomycin (Pen/Strep; 100 U/mL and 100 μg/mL, respectively), and 2 mM L-glutamine at 37 °C in a humidified 5% CO_2_ incubator. CHO-K1 cells were maintained in Ham’s F-12 (Gibco, New York, NY, USA) with 10% FBS, 1% Pen/Strep, and 2 mM L-glutamine under identical conditions. Cells were passaged at ~80–85% confluence using 0.25% trypsin-EDTA (Gibco, New York, NY, USA) and used between passages 5–15 [40]. TRPV1-transfected CHO cells (CHO-TRPV1) were generated by transient transfection as described in Section 4.2. All cell lines were regularly tested for mycoplasma contamination using a PCR-based MycoAlert detection kit (Lonza, Basel, Switzerland) and found to be negative throughout the experimental period.

### 4.2. TRPV1 Plasmid and Transient Transfection (CHO Cells)

CHO-K1 cells were transiently transfected with pcDNA3-EGFP-TRPV1 (full-length TRPV1) using TransFast Transfection Reagent (Promega, WI, USA) following the manufacturer’s instructions. Briefly, for each T25 flask at ~80% confluence, 5 μg plasmid DNA was diluted in 2 mL serum-free F-12; TransFast was added at a 3:1 (reagent: DNA; μL:μg) ratio, mixed, and incubated 15 min at room temperature. Culture medium was replaced with the DNA–reagent mixture for 1 h, then 4 mL complete medium was added and cells were incubated 48 h (37 °C, 5% CO_2_). Transfection efficiency was verified by EGFP fluorescence (Ex 488 nm/Em 509 nm) on an inverted fluorescence microscope (CLSFM, LSM-800, Zeiss, Oberkochen, Germany). Unless otherwise stated, CHO-TRPV1 denotes CHO cells 48–72 h post-transfection [21]. All cell lines were regularly tested for mycoplasma contamination using a PCR-based MycoAlert detection kit (Lonza, Basel, Switzerland) and found to be negative throughout the experimental period.

### 4.3. Reagents and Treatments

Cobalt (II) chloride hexahydrate (CoCl_2_·6H_2_O; Sigma-Aldrich, Burlington, MA, USA) was prepared fresh in sterile water. 17β-estradiol (E_2_; Sigma-Aldrich, Burlington, MA, USA) was dissolved in ethanol (EtOH) as 10 mM stock; capsaicin (CAP; Sigma-Aldrich, Burlington, MA, USA) and capsazepine (CPZ; Tocris, Bristol, UK) were dissolved in DMSO. Final vehicle concentrations were ≤0.1% (*v*/*v*) in all conditions.

### 4.4. Experimental Design

To clarify the experimental rationale and overall structure, all assays were designed to allow mechanistic comparison between estrogenic and hypoxic responses. The following points summarize the study design and its rationale in detail.

(i) Group structure and rationale: Four experimental conditions were established—Normoxia, E_2_, Hypoxia, and Hypoxia + E_2_ to distinguish ER-dependent and ER-independent effects on HIF-1α and TRPV1. This design allows direct mechanistic comparison between estrogenic modulation and hypoxic induction across both ERα(+) MCF-7 and ERα(–) CHO-TRPV1 cells, ensuring internal control within the same assay framework. Each group was processed under identical culture conditions and time frames to minimize variability arising from incubation duration or media composition. The inclusion of both ERα-positive and ERα-negative backgrounds was deliberate, as it provides a clear internal validation of whether estrogen exerts its effect through receptor-mediated signaling or through indirect modulation of hypoxia-driven pathways. In both cell types, parallel control groups were maintained to capture basal HIF-1α and TRPV1 levels under normoxic conditions, thereby establishing a baseline for relative quantification.
(ii) Biological replicates, randomization, and statistical power: All experiments were performed with at least three independent biological replicates, each including two technical repeats per assay to ensure reproducibility. Samples within each replicate were randomized across wells and measurement sessions to minimize operator bias. Each experiment was designed with adequate biological replication and statistical robustness, as detailed in Section 4.7, ensuring that observed differences represent genuine biological effects rather than procedural variability. This approach minimizes both Type I and Type II errors and increases the reliability of the statistical outcomes.
(iii) Validation of TRPV1 dependence: To strengthen mechanistic inference, capsazepine (10 μM, 10 min pre-incubation) was applied in Ca^2+^ signaling experiments to confirm TRPV1-specific Ca^2+^ influx. The suppression of Ca^2+^ elevation by capsazepine under both normoxia and hypoxia demonstrates that the observed effect is TRPV1-mediated. This pharmacological validation step serves as an internal functional control and verifies that any modulation of calcium signaling by hypoxia or estrogen arises from TRPV1 activity rather than from non-specific membrane leakage or unrelated channels. Furthermore, capsaicin was applied acutely (1 μM) to ensure that TRPV1 remained responsive within the physiological activation range, avoiding desensitization or cytotoxic overstimulation.
(iv) Temporal and experimental consistency: All treatments were standardized to a 24 h exposure window to capture early transcriptional and functional responses to hypoxia while preventing secondary adaptive effects related to prolonged stress. Experimental timing, reagent preparation, and imaging parameters were synchronized across replicates to maintain comparability between assays. All reagents were prepared freshly for each experiment, and identical passage ranges (5–15) were used to minimize cell line drift.
(v) Limitations and planned extensions: As now stated in the revised Discussion, the current design establishes a mechanistic relationship between hypoxia, HIF-1α, and TRPV1 activation, but genetic validation (TRPV1 siRNA or CRISPR silencing) and live-cell imaging of TRPV1 localization under hypoxia are planned to be conducted in future studies. Additionally, the inclusion of HIF-1α inhibitors or ERα modulators (e.g., ICI 182,780) is planned to further confirm the directional hierarchy of this signaling pathway. These next-step validations are planned to expand the current pharmacological framework into a fully integrated molecular model.

Unless indicated, treatments were applied for 24 h before assays:

- Control (Normoxia): vehicle only.
- E_2_: 10 nM 17β-estradiol (E_2_), a concentration within the physiological range commonly used in cell culture studies [22].
- Hypoxia (CoCl_2_): 200 μM CoCl_2_, 24 h [41,42].
- Hypoxia + E_2_: 200 μM CoCl_2_ + 10 nM E_2_ (added simultaneously).

For Ca^2+^ flux assays, acute pharmacology was applied on-line: CAP (1 μM, unless stated) to activate TRPV1; CPZ (10 μM, 10 min pre-incubation) to antagonize TRPV1. Where indicated, a CAP-free trace was recorded first to establish baseline.

### 4.5. Determination of Intracellular Free Ca^2+^ Concentration ([Ca^2+^]ᵢ)

To determine [Ca^2+^]ᵢ, CHO and MCF-7 cells were harvested, washed, and gently resuspended in HEPES-buffered saline (HBS; in mM: NaCl 140, KCl 4.7, CaCl_2_ 1.2, MgCl_2_ 1.1, d-glucose 10, HEPES 10; pH 7.4) at a final density of 2 × 10^6^ cells/mL. Cells were loaded with 4 μM Fura-2 AM (Invitrogen, Carlsbad, CA) for 45 min at 37 °C in the dark with gentle shaking. After loading, cells were briefly washed and allowed to undergo a 10 min de-esterification period in dye-free HBS at 37 °C to ensure complete hydrolysis of the AM ester before Ca^2+^ signaling. This step minimizes cytosolic background fluorescence and improves signal stability. Subsequently, cells were equilibrated for an additional 15 min in dye-free HBS before measurement. Fluorescence was recorded at 37 °C from 2 mL aliquots of magnetically stirred cell suspension using a spectrofluorometer (Varian Cary Eclipse, Sydney, Australia) with dual excitation at 340/380 nm and emission at 505 nm. Changes in [Ca^2+^]ᵢ were expressed as the F340/F380 ratio and calibrated according to the Grynkiewicz method [43]. For quantification, both the peak Δ[Ca^2+^]ᵢ and the 150 s integral after agonist addition were analyzed. In all groups, CAP (1 μM) was applied at t = 60 s to stimulate TRPV1-mediated Ca^2+^ entry. Where indicated, cells were pre-incubated with the TRPV1 antagonist capsazepine (CPZ, 10 μM, 10 min) prior to recording. [Ca^2+^]ᵢ values were expressed in nM, and data acquisition was performed at 1 Hz as described previously [40].

### 4.6. Western Blotting

For all experimental groups, Western blot analyses were performed following a standard procedure. Cells (MCF-7 and CHO-TRPV1) were rinsed with ice-cold PBS and lysed on ice using RIPA buffer (50 mM Tris-HCl, pH 7.5; 150 mM NaCl; 1% NP-40; 0.5% sodium deoxycholate; 0.1% SDS) supplemented with protease and phosphatase inhibitors (Roche, Mannheim, Germany). Lysates were clarified by centrifugation at 16,000× *g* for 20 min at 4 °C, and protein concentrations were determined using the Bradford assay at 595 nm. Equal amounts of protein (30 μg) from each sample were loaded on 8–12% SDS–polyacrylamide gels, electrophoresed, and transferred to nitrocellulose membranes. Membranes were blocked for 1 h at room temperature with 5% non-fat dry milk in Tris-buffered saline containing 0.1% Tween-20 (TBST). Blots were then incubated overnight at 4 °C with primary antibodies: anti-HIF-1α (1:1000; Cell Signaling Technology, Danvers, MA, USA), anti-TRPV1 (1:1000; Cell Signaling Technology, Danvers, MA, USA), and anti-β-actin (1:5000; Proteintech, Chicago, IL, USA) as the loading control. After TBST washes, membranes were incubated for 1 h at room temperature with HRP-conjugated secondary antibodies (1:5000; GE Healthcare, Amersham, UK). Protein bands were visualized using an enhanced chemiluminescence (ECL) substrate (Millipore Luminata Forte, Burlington, MA, USA) and imaged with a Syngene G: BOX system. Protein bands were visualized using enhanced chemiluminescence (ECL) and quantified by densitometry in ImageJ (version 1.54f, National Institutes of Health, Bethesda, MD, USA) after normalization to β-actin. Densitometric analyses were performed using ImageJ software, and protein expression levels were normalized to β-actin and expressed relative to control conditions [44].

### 4.7. Statistics

All data are expressed as mean ± SEM from at least three independent biological replicates, each including a minimum of two technical repeats. Normality and homogeneity of variance were verified using the Shapiro–Wilk and Levene tests, respectively. For group comparisons, one-way ANOVA (factor: treatment) or two-way ANOVA (factors: treatment × cell line) was applied as appropriate, followed by Tukey’s multiple comparisons test. A two-tailed *p* value < 0.05 was considered statistically significant. Statistical analyses were conducted using GraphPad Prism 10 (GraphPad Software, San Diego, CA, USA) and R version 4.3.1 (R Foundation for Statistical Computing, Vienna, Austria).

### 4.8. Reproducibility and Reporting

All reagents included catalog numbers and lot tracking; incubation times, temperatures, and concentrations are stated explicitly above to facilitate replication. No randomization or blinding was applied to in vitro conditions; all assays were pre-planned, and exclusion criteria (poor loading, viability <90%, or transfection efficiency <40% in CHO-TRPV1) were defined a priori.

### 4.9. Ethics Statement

This study used established human and hamster cell lines only; no human participants or animals were involved, and ethics approval was not required.

## 5. Conclusions

In summary, this study demonstrates that hypoxia stabilizes HIF-1α, increases TRPV1 expression, and amplifies Ca^2+^ influx in both MCF-7 and CHO-TRPV1 cells, confirming a functional coupling between HIF-1α activation and TRPV1 channel responsiveness. Importantly, in ERα-positive MCF-7 cells, E_2_ significantly counteracted the hypoxia-induced up-regulation of both HIF-1α and TRPV1, thereby limiting the extent of Ca^2+^ accumulation. In contrast, ERα-deficient CHO-TRPV1 cells displayed a purely channel-driven response, as E_2_ had no detectable effect on Ca^2+^ influx or TRPV1 expression. These observations establish two mechanistically distinct modes within the hypoxia, HIF-1α, TRPV1 and Ca^2+^ axis: an ER-dependent regulatory pathway, in which estrogen signaling suppresses excessive calcium entry, and an ER-independent pathway, in which TRPV1 activation proceeds unrestrained by hormonal modulation.

The data further suggest that E_2_-mediated inhibition of HIF-1α may represent a molecular link between estrogen signaling and calcium homeostasis under hypoxic stress. This dual control mechanism provides an experimental basis for understanding how ERα status influences the cellular susceptibility to hypoxia-induced calcium overload and oxidative injury.

Biologically and clinically, these findings identify TRPV1 as a context-specific effector of hypoxic signaling that may contribute to endocrine resistance and calcium-dependent cell injury. Future work should explore whether pharmacological TRPV1 antagonists, antioxidant compounds, or HIF/ERRα modulators can synergistically mitigate hypoxia-driven Ca^2+^ dysregulation in ERα-positive tumor models. Collectively, the present results support a coherent mechanistic framework linking hypoxia, HIF-1α stabilization, and TRPV1-mediated Ca^2+^ dynamics, with direct implications for therapeutic targeting in estrogen-responsive cancers.

Limitations:

Although the current study provides clear evidence of estrogenic modulation of the hypoxia, HIF-1α and TRPV1 axis, it is limited to in vitro observations in two cell models. The absence of genetic TRPV1 silencing or in vivo validation restricts the extent to which these findings can be generalized. Future studies incorporating TRPV1 knockdown and in vivo hypoxia models will be required to confirm the broader physiological relevance of this pathway.

## Data Availability

The original contributions presented in this study are included in the article. Further inquiries can be directed to the corresponding author.
